# On the Constitutional and Local Effects of Disease of the Supra-renal Capsules

**Published:** 1859-03

**Authors:** 


### NORTH AMERICAN
### MEDICO-CHIRURGICAL REVIEW.

MARCH, 1859.

### Analytical and Critical Reviews.

ART. I.—*On the Constitutional and Local Effects of Disease of the Supra-renal Capsules.* By THOMAS ADDISON, M.D., Senior Physician to Guy's Hospital, London. 4to., pp. 43. With Plates.

*Series of Cases Illustrating the Connection between Bronzed Skin and Disease of the Supra-renal Capsules. With Analyses and Comments.* By JONATHAN HUTCHINSON, (in *Medical Times and Gazette*, vols. xi., xii., and xiii., 1855 and 1856.)

*Recherches Experimentales sur la Physiologie et la Pathologie des Capsules Surrenales.* Par le DOCTEUR E. BROWN-SÉQUARD, (*Archives Gênerales de Medecine*, Oct. et Nov., 1856.)

*Nouvelles Recherches Experimentales sur l'Importance des Fonctions des Capsules Surrenales.* Par le Même, (*Journal de la Physiologie des Hommes et des Animaux.* Publié sous la Direction de DOCTEUR E. BROWN-SÉQUARD, Janvier, 1858.)

*An Experimental Inquiry into the Functions of the Supra-renal Capsules, and their Supposed Connection with Bronzed Skin.* By GEORGE HARLEY, M.D., F.R.S., (*British and Foreign Medico-Chir. Review.* January and April, 1858.)

*The Sunburnt Appearance of the Skin as an Early Diagnostic Symptom of Supra-renal Capsule Disease.* By ISAAC E. TAYLOR, M.D., Physician to the Bellevue Hospital, New York. With Colored Illustrations. (Reprinted from the *New York Journal of Medicine,*) pp. 21. New York, 1856.

IT is not quite four years since Dr. Addison, the eminent senior physician to Guy's Hospital, startled the whole medical world by announcing, on evidence of which he was the first to see the bearing, the important fact that morbid changes in the hitherto-thought functionless capsulæ renales, were attended by disease of an exhausting and generally fatal nature, one of the most characteristic symptoms of which is a peculiar brown, or a bronzed skin. The other leading symptoms, as described by its discoverer, "are anæmia, general languor and debility, remarkable feebleness of the heart's action, and irritability of the stomach." The hesitation and doubt, if not incredulity, with which Dr. Addison's views were received in different quarters need not excite surprise. This state of mind was very natural, when we look at it in connection with the almost universal disbelief in the supra-renal capsules exercising any function whatever in the adult state, or, indeed, in any period of extra-uterine life. Whatever offices they might be conjectured to perform were, it was admitted by common consent, associated with fœtal existence. Not that a shade of doubt could be supposed to hang over the statements made by Dr. Addison, both as regards the disease and the *post-mortem* lesions which he describes. The dissent was to the extent of viewing as cause and effect, or as necessarily connected, the two series of phenomena; those in the living and those in the dead body, in these circumstances. It might have been asked, whether the fatal result was not due to lesions and degenerations in other organs, or in the blood itself? Fortunately this state of suspense was designed to be of short duration—and to Dr. Addison must be awarded the twofold merit: first, of recognizing and describing a new disease in connection with textural changes in an organ previously neglected, and deemed to be of no note; and, secondly, of putting forth his work, to use his own modest language in his preface, "as a first and feeble step toward an inquiry into the functions and influence of these organs, suggested by pathology."

The appeal thus made met with a ready response from one whose rising fame and already large contributions to physiological science are known in both hemispheres. Doctor E. Brown-Séquard, to whom we now refer, had his attention drawn to the subject soon after the issue of Dr. Addison's work, and he lost no time in instituting a series of experiments, with a view of ascertaining the functions of the supra-renal capsules. In availing ourselves of the researches of Dr. Brown-Séquard, as they are described in the journals designated at the head of this paper, and of those of Dr. Harley, we shall waive the chronological order in which the different topics connected with the whole subject have come up in the way of discovery and discussion, and shall begin with a succinct account of what is now known of the physiology of the supra-renal capsules, and then turn to account the pathological discovery of Dr. Addison, and the confirmatory observations of Mr. Hutchinson. By adopting this plan we hope to be able to exhibit a connected view of the probable relation of these bodies to the rest of the organism, and thus supply a want, which, we will venture to say, is felt by a large body of the profession.

In a paper in his *Journal of Physiology,* Dr. Brown-Séquard adverts to a remarkable fact published by him in 1857. It is, that a section of the lateral half of the spinal marrow at the dorsal and lumbar regions, in a Guinea-pig, was followed, at first, by congestion, and, after many months, by hypertrophy of the two supra-renal capsules. This singular connection between the spinal marrow and these small organs is the more worthy of notice when it shall be shown, as Dr. Brown-Séquard proposes to do in another memoir, that in some cases of fracture of the spine a noticeable congestion of the supra-renal capsules must be ranked among the circumstances by which death is hastened. In his first investigations, made expressly on the subject, a notice of which is contained in the *Comptes Rendus* of the session of the Academy of Sciences, in 1856, he declared that in all the animals from which he had removed the supra-renal capsules, or even one of them, death had rapidly supervened. The author also announced the fact that death occurred too soon after the ablation of the capsules to allow of our believing that it was due exclusively to a lesion of the kidneys, the liver, or the great sympathetic nerve. He remarked that the blood of the animals thus deprived of their supra-renal capsules, seemed to be charged with a toxical element. At least it was found that if the blood of rabbits, taken at the moment of their death, following ablation of these bodies, was injected into the veins of other rabbits, which had lost one of their capsules a few hours previously, it gave rise to the speedy death of these latter; whereas, on the other hand, the blood of a rabbit in good health, injected into the veins of a rabbit in its dying agony, caused its resuscitation for a period of some hours' duration. He added, moreover, that ablation of the kidneys does not destroy life so soon as that of the supra-renal capsules. M. Gratiolet, it may as well be mentioned at this time, wrote to the Academy, telling of his having made some experiments on Guinea-pigs, from which it appeared that excision of the left supra-renal capsule was not always followed by death, while that of the right capsule was constantly fatal, in consequence of inflammation of the liver and peritoneum. At the next meeting of the Academy, Dr. Brown-Séquard repeated his conviction, that death in these cases was not owing to accidental inflammation, which, from its being slowly developed, could not attain a sufficient degree of intensity to cause death at the time when this event took place.

Not long after this, Dr. Brown-Séquard wrote an extended memoir,*

---

* Experimental Researches, etc., on the Physiology and Pathology of the Supra-renal Capsules.

which appeared in two successive numbers (October and November, 1856,) of the *Archives Générales.* He begins by referring to some publications of great merit which appeared before the noted work of Dr. Addison, but which were confined almost entirely to the anatomy and pathology of the supra-renal capsules. Among these he designates M. Rayer's summary account in 1837, and a valuable production by Alexander Ecker in 1846, containing excellent views on the microscopic anatomy of these organs. In the memoir which we now propose to analyze its author announces his intention of exhibiting a series of facts bearing on the physiological and pathological history of the supra-renal capsules, and to compare the phenomena observed in man with those which he witnessed in animals.

The long and almost universally accredited opinion, that the supra-renal capsules were organs exclusively, or chiefly, belonging to fœtal life, is subjected to the test of experiment by Dr. Brown-Séquard, and shown to be unfounded. Stress was laid on the earlier and proportionately more rapid growth of these glands than of the kidneys in the embryo. It is only at the tenth or the twelfth week of intra-uterine existence that the kidneys have acquired the size of the capsules. By the sixth month the kidneys are twice the size of the capsules, and their weight is to that of the latter as five to two. In the new-born infant the proportions of the two in weight are as three to one; and in the adults the capsules are only one-half of the weight of the kidneys. Even if we admit these facts, what, asks the author of the memoir, do they prove? Nothing more than that the functions of the supra-renal capsules begin sooner than those of the kidneys. But this inference only applies to the human subject, for in the other mammiferæ they are always, from the earlier period of embryonic life to adult age, smaller than the kidneys. If the supra-renal capsules were exclusively related to embryonic life they would disappear or become atrophied after birth; but this is so far from being the case that they acquire an increased weight and three times the volume in adult life beyond what they had at birth. At the latter date the capsules vary in weight from 32 to 64 grains; and in the former or adult period, from 105 grains to rather more than 3 drachms. Taking the kidneys as organs for comparison, Dr. Brown-Séquard found that in dogs, cats, and Guinea-pigs, these organs in females weighed seven to ten times more than those of their freshly born progeny, while the capsules of the former were to those of the latter, in weight, as eight to eleven. It is obvious then from these data, that organs which grow in so noticeable a manner, from birth to adult age, must be endowed with function; and that it is an error to speak of them as belonging exclusively to fœtal existence.

We learn from the experiments of Dr. Brown-Séquard, that the supra-renal capsules are endowed with a high degree of sensibility. This might have been expected from their abundant supply of nerves; to such a degree indeed that some authors have regarded them as a kind of nervous ganglions. The presence of nerve-cells in the interior of these organs would seem to favor this belief, were it not ascertained by Nugel, Bardeleben, Ecker, and Frey, that they are glands. It is, nevertheless, certain, that they are more largely supplied with nerves than any other organ in the mammiferous animals; although this is not the case in the other vertebrate classes. The discovery of glandular vesicles in the supra-renal capsules of the four classes of the vertebratæ, has caused these organs to be definitively ranked among the ones which are now called sanguineous or vascular glands. The author is able to affirm, in relation to their nervous endowment, that the supra-renal capsules in the dog, cat, rabbit, and Guinea-pig, contain some fibres of Remak, and very seldom fibres of double circuit; but, on the other hand, very fine nervous filaments (the sympathetic fibres of Bidda and Volkmann) abound in them. The sensibility of the organs in question is incontestably greater than that of the other abdominal viscera in these animals. It is very acute in the rabbit, and nearly as great in the cat, but less in the dog and Guinea-pig.

Dr. Brown-Séquard ascertained by experiments that the mean period of survival, after the simultaneous ablation of the two supra-renal capsules, was nine hours and some minutes for rabbits, fourteen hours for adult cats and dogs, eight hours for two mice, thirteen hours for eleven adult Guinea-pigs, twenty-three and a half hours for four young Guinea-pigs, and thirty-seven hours for very young dogs and cats—which would give a total mean of seventeen and a half hours. A repetition of these experiments, as described in his last memoir, *New Researches*, etc., gave nearly identical results. He witnessed the survival of dogs and Guinea-pigs, after the ablation of the right supra-renal capsule, that which, according to M. Gratiolet, could not be done without the supervention of a fatal hepatitis and peritonitis. Dr. Brown-Séquard had seen death take place with a frequency and a rapidity, after the ablation of a single capsule, (the right or the left one,) especially in rabbits, to convince him that, even in this case, death was owing to the absence of the excised organ.

Among the very interesting symptoms which were observed after the ablation of the two capsules, the author designates, in a more particular manner, weakness and invariable disorder of the respiration and the circulation. The force of the heart's movements is always diminished, as is also, at first, the frequency of its contractions; but, after awhile, when the breathing, from being at first more active, becomes disturbed and slow, the heart beats quicker. It may be added, that during all this time there is no *fever*. With two exceptions, the animals which had lost their suprarenal capsules refused to take food. In the last period of life there is usually delirium, and very often convulsions occur, which resemble those caused by strychnia. Sometimes the convulsions are epileptiform, with a tendency to roll over on the left side, if the right supra-renal capsule have been removed, and *e contrario* as relates to the other. In rabbits there was often noticed a turning round.

The experimenter found in the blood of the animals, which had been deprived of their capsules more pigment than is commonly met with, and often this pigment was in laminæ, much larger than the diameter of the capillaries of the encephalon. He ascertained that the formation of the blood crystals, differing in some particulars from those which are found in the normal blood, takes place, often spontaneously, and with rapidity, and in great abundance, in the blood extracted from the vessels of animals deprived of their supra-renal capsules.

Dr. Brown-Séquard has observed a disease very prevalent among rabbits in Paris, which would seem to be always fatal, and in a very brief period too. He endeavors to show that this disease is connected with a redundant production of pigment and a consecutive inflammation of the supra-renal capsules,—and that its symptoms are analogous to those caused by ablation of the capsules, as both are to those of the disease described by Addison. On comparing the phenomena which the author noticed in animals, with those observed by Dr. Addison and others, in the human subject, it appears that the cessation of the functions of the supra-renal capsules is followed by an accumulation of pigment in the blood of the former, and the skin of the latter. Hence there is reason to suppose that one of the functions of the supra-renal capsules consists in modifying a substance endowed with the property of being transformed into pigment.

The author, in his *New Researches*, etc., published in his own journal, replies to M. Philipeaux's and Dr. Harley's inferences from experiments made by them, and which to some extent militate against the views which he had advanced, and of which we have just given a synopsis. M. Philipeaux had at first announced, in a memoir addressed to the Academy of Sciences, that he had seen four albino rats survive the ablation of the supra-renal capsules. In a subsequent communication, however, he reports the death of three of those animals, at periods of, respectively, nine, twenty-three, and thirty-four days, after the excision of the left supra-renal capsule; the right having been removed some weeks previously. He attributes these deaths, but without good cause, to cold. When death does occur after the operation, M. Philipeaux thinks it is owing to peritonitis, hepatitis, or to intestinal hernia. To this, Dr. Brown-Séquard replies, by showing that whereas lesions of the peritoneum, liver, renal veins, and vena cava do not destroy life, in many instances under twenty hours, and sometimes three weeks, a removal of the supra-renal capsules, accompanied by much less local irritation, is followed by death in seven to fourteen hours. He added that, very probably, other glands, such as the thyroid and the thymus, might supply the functional rôle of the capsules, when these are wanting. He concedes, at the same time, that animals may survive, as shown by the experiments of MM. Philipeaux, Martin Magron, and Dr. Harley, the ablation of their supra-renal capsules.

The whole question, as far as relates to the deductions drawn from experiments, is exhibited by Dr. Brown-Séquard under the following aspects. In the first place, all the physiologists who have repeated his experiments have noticed, as he had, that death invariably takes place, whatever may be the animal, after the *simultaneous* ablation of the two supra-renal capsules. It appears that even albino rats, under these circumstances, die, as well as rabbits, cats, dogs, Guinea-pigs, mice, rats which are not albinos, pigeons, etc. In the second place, even when the ablation of a single capsule is practiced, a certain number of days after the other had been removed, there is no instance hitherto observed of definitive survival except in albino animals; that is to say, animals without pigment. Now the author has pointed out, as one of the causes of death after the ablation of the supra-renal capsules of animals which are not albinos, the presence in the blood of plates of pigment too large to allow of their passage through the small capillaries of the encephalon, and which give rise to hemorrhage or defective circulation in this organ. Even if he is mistaken in supposing this to be a cause of death after the removal of the capsules, it is nevertheless certain that these little organs bear some relation to the production of a dark pigment; for, in more than sixty-five cases collected within the last few years, there has been noticed in the human subject the coexistence of a deposit of pigment in the skin, and a great change in the structure of the supra-renal capsules. There is, then, a casuality relation between the two facts: absence of the functions of these capsules, and increase of the dark pigment. If albino rats do not die after the ablation of the two supra-renal capsules, it would seem to be an important proof, in addition to those furnished by Dr. Brown-Séquard, that it is in part owing to an accumulation of dark pigment that death takes place in animals which are not albinos, when they are deprived of their glands.

Our attention is directed to a fact of some interest in the present inquiry by Dr. Brown-Séquard. It is the suddenness of the death of animals which seemed to have survived all the consequences of the ablation of the supra-renal capsules, and were in good health. Cases of this nature occurred in the experience of MM. Philipeaux, Martin

Magron, Dr. Harley, and the author himself. There are marked differences in the toleration, it might be called, of the removal of the capsules, or in the periods of the survival after the operation, depending on the age and species of the animal. Cats survive longer than dogs, rabbits, or Guinea-pigs; and the very young survive for a decidedly longer period than adults. A repetition of former experiments authorizes their author to repeat in this memoir what he had advanced on a former occasion, viz., that animals live much longer after an ablation of their kidneys than of their supra-renal capsules.

Dr. Brown-Séquard draws, as well from his own experiments as from those of physiologists who have disputed his views, the following conclusions:—

1. The functions of the supra-renal capsules seem to be essential to the life of animals which are not albinos.

2. The *immediate* and *complete* suppression of these functions gives rise to speedy death.

3. The *gradual* suppression of these functions is followed by death, at the very latest, after a few months, and in certain species of animals after a few days.

4. The simultaneous ablation of the two supra-renal capsules brings with it, in general, death sooner than the removal of the two kidneys.

5. If certain albino animals seem to be capable of surviving definitively the ablation of the supra-renal capsules, this fact corroborates the opinion which he had previously announced, that one of the chief causes of death in animals not albinos, after the loss of these small glands, consists in the accumulation of pigment.

Doctor Harley, in his *Experimental Inquiry*, treats of—1. The histology of the supra-renal capsules; 2. Their chemical composition; 3. The effects of their removal from healthy animals; and 4. Their pathology. We cannot, for want of space, follow the author in his morphological description, and extracts would fail to convey the requisite knowledge on this point. Doctor Harley agrees with Doctor Brown-Séquard in believing that the supra-renal capsules ought not be regarded as fœtal organs, since they continue to increase in size, in a certain ratio, after birth. We pass on to a brief notice of the chemical division of the subject, respecting which our information is still meagre. The discovery, by M. Vulpian, of a beautiful rose-color produced in the medullary substance of the capsules has not led to any conclusion respecting its import. Nor has the detection, by MM. Vulpian and Cabrez, of hippuric and taurocholic acids in the supra-renal capsules of herbivorous animals been productive of more fruitful results, as these substances have been found abundantly in different parts of the animal body.

Doctor Harley's investigations into the physiology of the supra-renal capsules have led him to opposite conclusions to those reached by Doctor Brown-Séquard. Doctor Harley rendered the animals insensible either by the smoke of the common puff-ball, or by ether. He operated on an adult cat, by removing, without hemorrhage, the right supra-renal capsule. The animal lived nine days after the operation, and was apparently in good health up to the eighth day. It was then noticed that she had lost flesh. The only morbid appearance, on examination, was inflammation with slight opacity of the peritoneum on the operated side. As one of the objects of Doctor Harley's extirpating the capsules was to ascertain if their removal would be attended by any change of color in the skin or its appendages, he took for his experiment a nearly white cat, both the supra-renal capsules of which were extracted at one time. Twenty-four hours after the operation the animal was found dead. "On *post-mortem* examination, some lymph was found effused upon one of the kidneys. There was no pus, but all the other signs of peritonitis." Dr. Harley usually deemed it necessary when operating upon animals, like the cat and dog, to ligate a vein of considerable size, which crosses directly over the middle of the capsule. Thinking that the injury done to this vessel might have caused phlebitis, he put a ligature round half of the left supra-renal capsule of a small white coach-dog, avoiding the vein altogether, and cut away the other half of the organ. Contrary to what might have been expected from Dr. Brown-Séquard's assertion, as the result of his experiments, that mere puncture of the organ is rapidly fatal, this dog, the next morning, came running to the door of the room in which he was confined, frisked about, and was hungry for his food. Six days afterwards, however, he was much less lively: on the ninth day he refused to eat, and on the twelfth day he died. A *post-mortem* examination revealed extensive and intense signs of peritonitis. "The intestines were glued together, and flakes of lymph abounded all over the abdominal walls. A large abscess, surrounded by an adventitious membrane, two lines in thickness, occupied the left hypochondriac region. It involved the lower half of the spleen, and rested upon the kidney. It does not, however, include this latter organ." The proximate cause of death was clear in this case, whatever opinions may be entertained as to its being consecutive on injury done to the supra-renal capsule, owing to the presence of the ligature, or to damage done to neighboring parts after the operation. Experiments showed that death will follow from another cause of injury in adjoining parts besides mere removal of the capsule. Dr. Harley next applied himself to the removal of the organ without mutilation of neighboring parts; and he performed the operation on a large tom-cat, by enucleating both the capsules at the same time. In this case he was astonished to find that the capsules were hard as a stone, and on making a section of them, a considerable portion of the medullary, as well as of the cortical substance, was replaced by a calcareous deposit, which consisted chiefly of carbonate of lime. The remaining portions of the gland contained so much fibrous tissue that the normal structure might be said to have entirely disappeared. Much to his surprise, Dr. Harley found the cat dead on the next morning after the operation, notwithstanding that this had been performed with the utmost facility, and there had been no hemorrhage or any apparent injury done to the surrounding parts, except the tearing through of the vessels and nerves. *Post-mortem* examination failed to reveal the mystery. "Nothing, absolutely nothing, was to be seen but a very trifling attempt at peritonitis." The author was very naturally indisposed to believe that the death could be owing to a removal of the supra-renal capsules, as the calcareous degeneration to which they had been subjected must, for a length of time previously, have destroyed their function, whatever it may be.

Dismissing, as unworthy of attention, the effects of tearing the blood-vessels and lymphatics, Dr. Harley takes up the question of the consequences of tearing across the nerves going to the supra-renal capsules. These, though small, are, as described in Quain's Anatomy, which he quotes, "exceedingly numerous. They are derived from the solar plexus of the sympathetic, and from the renal plexus. According to Bergmann, some filaments come from the phrenic and pneumogastric nerves. They are made up mainly of dark-colored white fibres, of different sizes, and they have many small ganglia upon them." The reader will remember the very abundant nervous supply to these bodies, described by Dr. Brown-Séquard in a preceding page. Dr. Harley asks, "what would be the result of mutilating the above-named nerves, independent of the removal of the supra-renal capsules? It is an established fact that instant death frequently follows upon sudden injury to the solar plexus. A blow on the epigastrium is often sufficient to prove rapidly fatal to life." Dr. Brown-Séquard has shown that mere pricking or cutting of the semilunar ganglion proves fatal to rabbits in the course of thirty hours, while division of the sympathetic, in the neighborhood of the kidney, causes death within twenty-three hours after the operation. Ludwig and Hoffer found that a section of the great splanchnic killed animals in the space of one or two days. "Such being the case, need we hesitate," argues Dr. Harley, "in the absence of all other proof, as to the cause of death in the above-mentioned experiments, to regard it as probable that the animal died from the injury done, in tearing out the capsules, to the ganglionic system of nerves?"

Dr. Harley regards the extirpation of the right capsule as more dangerous than that of the left, owing to the former being much more intimately connected with the surrounding parts, and especially to its lying closer to the semilunar ganglion than the latter; and hence greater injury is likely to be done to the sympathetic system of nerves in the extirpation of the right than of the left supra-renal capsule. Dr. Harley performed the operation of removing at first, the right, and nine days afterwards, the left supra-renal capsule, of an albino rat. The animal died twenty-five days after the extirpation of the right, and sixteen days after the removal of the left of these glands. "The exterior of the body was carefully examined, but no discoloration of the skin could be detected." On opening the abdomen the intestine looked like a gelatinous mass, and their walls were remarkably thin and transparent. The right half of the stomach was in a similar condition Dr. Harley believes that the degeneration of the stomach and intestines was owing to the injury done to the ganglionic system of nerves, especially to those branches supplied to the capsules by the solar plexus. We do not learn that a similar degeneration followed removal of the capsules, even where greater injury was done to the nerves and surrounding parts than in the operation now noticed. The animal rapidly recovered after the extirpation of the right capsule, although it was attended with the most difficulty. The left capsule, we are told by the operator, was extracted with the greatest facility. In another case, of a piebald rat, the operation, which consisted in the ablation of the right supra-renal capsule, was a tedious and disagreeable one, in consequence of the animal not having been rendered sufficiently insensible. As it lasted several minutes, there was little hopes of the animal surviving, but, contrary to expectation, it made a tolerably rapid recovery, and in six weeks, as it was in excellent condition, the other capsule was removed. In this case, as in that of a cat on which a similar operation had been performed, Dr. Harley was unable to find any very marked difference in size or appearance, as might, *a priori*, have been supposed; the left having sufficed during a period of six weeks to perform the function of the two capsules.

With regard to the connection of the supra-renal capsular function and bronzing of the skin, the author contents himself, for the present, with remarking that in the case of this piebald rat no increase of pigment was observed to take place.

The following observations of Dr. Harley militate strongly against the conclusions reached by Dr. Brown-Séquard, on the question of the survivals of animals after the loss of the supra-renal capsules. "Rats not only survive after extirpation of the supra-renal capsules, but even become strong and healthy after the removal of the spleen also. I have, at present, in my possession, some animals from which the spleen and suprarenal capsules were removed when they were a month old. Yet, notwithstanding this, they have grown up without any apparent modification of their functions having taken place, and are now fine specimens of the well developed adult animal." M. Philipeaux has been more successful; he has "removed the spleen, the supra-renal capsules, and the thyroid glands from the same animal, and it has afterwards recovered."

Dr. Harley thinks that from the experiments detailed by him, the chief of which we have introduced to the knowledge of our readers, the following conclusions may be drawn:—

"1st. The supra-renal capsules are not solely fœtal organs.

"2d. The supra-renal capsules are not absolutely essential to life.

"3d. The removal of the right is more generally fatal than the removal of the left capsule.

"4th. That convulsions do not necessarily follow the removal of the capsules.*

"5th. That the absence of their functions (in rats) is attended neither by great emaciation nor debility.

"6th. That when death follows upon the extirpation of the supra-renal bodies, it is in most cases the consequence of the injury done to the neighboring tissues, perhaps most frequently the mutilation of the ganglionic system of nerves.

"7th. Absence of the function of the supra-renal bodies is not proved to have any special effect in arresting the transformation of hæmatin, or in increasing the formation of blood crystals.

"8th. The suppression of the supra-renal capsular function is not attended by an increased deposit of pigment in the skin or its appendages (in rats.)

"9th. The problem of the connection of a bronzed skin and supra-renal capsular disease is more likely to be solved in the dead-house than in the physiological laboratory."

So far as inquiry into the subject has yet gone, the last conclusion of Dr. Harley would seem to be justified by a full and impartial survey of the premises, notwithstanding the more favorable light in which the physiology resting on vivisections on animals is placed by Dr. Brown-Séquard, to illustrate the pathology of the disease in the human body, as described by Dr. Addison. To this gentleman's clinical observations, then, we turn for information on a topic, which is at once new, curious, and instructive:—

"As a preface to my subject," writes Dr. Addison, "it may not be altogether without interest or unprofitable, to give a brief narrative of the circumstances and observations by which I have been led to my present convictions.

"For a long period I had, from time to time, met with a very remarkable form

* In a letter dated November 15, [1857,] Professor Virchow informs me that he has also extirpated the supra-renal capsules without having noticed the derangement of the central nervous system, mentioned by Dr. Brown-Séquard. I should add that I have seen two animals suffer from convulsions after extirpation of the capsules. One, a cat, died soon after having slight tetanic convulsions.

of general anæmia, occurring without any discoverable cause whatever; cases in which there had been no previous loss of blood, no exhausting diarrhœa, no chlorosis, no purpura, no renal, splenic, miasmatic, glandular, strumous, or malignant disease. Accordingly, in speaking of this form of anæmia in a clinical lecture, I, perhaps with little propriety, applied to it the term 'idiopathic,' to distinguish it from cases in which there existed more or less evidence of some of the usual causes or concomitants of the anæmic state.

"The disease presented in every instance the same general character, pursued a similar course, and with scarcely an exception, was followed, after a variable period, by the same fatal result. It occurs in both sexes generally, but not exclusively, beyond the middle period of life, and so far as I at present know, chiefly in persons of a somewhat large and bulky frame, and with a strongly marked tendency to the formation of fat. It makes its approach in so slow and insidious a manner, that the patient can hardly fix a date to his earliest feeling of that languor which is shortly to become so extreme. The countenance gets pale, the whites of the eyes become pearly, the general frame flabby rather than wasted; the pulse perhaps large but remarkably soft and compressible, and occasionally with a slight jerk, especially under the slightest excitement; there is an increasing indisposition to exertion, with an uncomfortable feeling of faintness or breathlessness in attempting it; the heart is readily made to palpitate; the whole surface of the body presents a blanched, smooth, and waxy appearance; the lips, gums, and tongue seem bloodless; the flabbiness of the solids increases; the appetite fails; extreme languor and faintness supervene; breathlessness and palpitation being produced by the most trifling exertion or emotion; some slight œdema is probably perceived about the ankles; the debility becomes extreme, the patient can no longer rise from his bed, the mind occasionally wanders, he falls into a prostrate and half torpid state, and at length expires; nevertheless to the very last, and after a sickness of perhaps several months' duration, the bulkiness of the general frame and the amount of obesity often present a most striking contrast to the failure and exhaustion observable in every other respect.

"With perhaps a single exception, the disease, in my own experience, resisted all remedial efforts, and sooner or later terminated fatally. On examining the bodies of such patients after death, I have failed to discover any organic lesion that could properly or reasonably be assigned as an adequate cause of such serious consequences; nevertheless from the disease having uniformly occurred in fat people, I was naturally led to entertain a suspicion that some form of fatty degeneration might have a share at least in its production; and I may observe that in the case last examined, the heart had undergone such a change, and that a portion of the semilunar ganglion and solar plexus, on being subjected to microscopic examination, was pronounced by Mr. Queckett to have passed into a corresponding condition. Whether any or all of these morbid changes are essentially concerned, as I believe they are, in giving rise to this very remarkable disease, future observation will probably decide.

"The cases having occurred prior to the publication of Dr. Bennet's interesting essay on 'Leucocythæmia,' it was not determined by microscopic examination whether there did or did not exist an excess of white corpuscles in the blood of such patients," pp. 2–4.

It was while seeking, but in vain, to throw some additional light upon this form of anæmia, that Dr. Addison stumbled, as he modestly expresses it, upon the curious facts which it is his more immediate object to make known to the profession in the short monograph which we are now turning to account for the benefit of our readers. Many stumble on facts, but for the most part put them to one side as of no interest, or, if retained, they are arranged among the *actæ curiosæ*, or help to form a string of party-colored beads, to be shown, occasionally, to an inquisitive friend. Dr. Addison, in a more philosophic spirit, noted the resemblance of the symptoms of several cases of disease, and then watched for any peculiarity of organic lesion on the bodies of those who sank under them. This peculiarity he found in alterations of the supra-renal capsules. One external and characteristic feature of the disease arrested his attention, viz.: discoloration of the skin, "sufficiently marked, indeed, as generally to have attracted the attention of the patient himself, or of the patient's friends."

"This discoloration pervades the whole surface of the body, but is commonly most strongly manifested on the face, neck, superior extremities, penis, and scrotum, and in the flexures of the axillæ, and around the navel. It may be said to present a dingy or smoky appearance, or various tints or shades of deep-amber, or chestnut-brown; and in one instance, the skin was so universally and so deeply darkened, that, but for the features, the patient might have been mistaken for a mulatto."

"In some cases, this discoloration occurs in patches, or perhaps, rather certain parts are much darker than others, so as to impart to the surface a mottled, or somewhat checkered appearance; and in one instance, there were, in the midst of this dark mottling, certain insular portions of the integument presenting a blanched or morbidly white appearance, either in consequence of those portions having remained altogether unaffected by the disease, and thereby contrasting strongly with the surrounding skin, or, as I believe, from an actual defect of coloring matter in these parts," p. 5–6.

The irregular distribution of pigment-cells is by no means limited to the integument, but is occasionally, also, manifest in some of the internal structures, as in the form of small black spots beneath the peritoneum of the mesentery and omentum—a form which in one instance presented itself on the skin of the abdomen.

"This singular discoloration usually increases with the advance of the disease, the anæmia, languor, failure of appetite, and feebleness of the heart become aggravated; a darkish streak usually appears upon the commissure of the lips; the body wastes, but without the extreme emaciation and dry harsh condition of the surface, so commonly observed in ordinary malignant diseases; the pulse becomes smaller and weaker, and without any special complaint of pain or uneasiness, the patient at length gradually sinks and expires."

The connection between lesion of the capsules and the symptoms, as regards degree in both, is thus described by the author :—

"When the lesion is acute and rapid, I believe the anæmia, prostration, and peculiar condition of the skin will present a corresponding character; and that whether acute or chronic, provided the lesion involve the entire structure of both organs, death will inevitably be the consequence."

Dr. Addison lays great stress on a correct and certain diagnosis, as the chief difficulty to be surmounted by further experience in this, as he believes, irremediable disease. At a very early period, when the capsules are less extensively diseased, the discoloration may doubtless be so slight and equivocal as to render the source of the anæmic condition uncertain. Here we must have recourse to negative evidence, and if we find, with marked anæmic disorder, an absence of its well-known sources, after an examination of the organs usually concerned, we may suspect with good reason the disease in question. Our belief in this effect will be increased if there be superadded a dark, dingy, or smoky-looking discoloration of the integument.

"It must, however, be observed that every tinge of yellow, or mere sallowness, throws a still greater doubt over the true nature of the case; and that the more decidedly the discoloration partakes of the character described, the stronger ought to be our impression as to the capsular origin of the disorder."

Nine cases are recorded by Dr. Addison, in which, during life, the characteristic symptoms already described were present, and on dissection of the bodies after death, the supra-renal capsules were found to be greatly altered; in one case by simple inflammation, in the others by strumous or malignant deposit. One of the cases, occurring in 1829, is taken from Dr. Bright's Reports. No suspicion was then entertained of the very dark color of the complexion and other symptoms being connected with the only marked organic change, that of the capsules, found on *post-mortem* examination. Portraits of some of the dark-skinned patients, and colored plates of the morbid changes of the capsules are added to the text of the remarkable monograph of Dr. Addison.

This gentleman has been gratified in his wish to attract the attention of the profession at large to the curious facts which he has brought before them, and to enlist their co-operation, so as to have the subject "properly examined and sifted, and the inquiry so extended as to suggest at least some interesting physiological speculations, if not still more important practical indications." As relates to the physiological part, we have seen how Dr. Brown-Séquard and other French experimenters, and Dr. Harley, of London, have responded to Dr. Addison's wish. It remains for us to show how ably he has been seconded in regard to the pathological indications by Mr. Jonathan Hutchinson, in successive papers furnished by this gentleman to the *Medical Times and Gazette* for 1855 and 1856, (vols. xi., xii., and xiii., New Series.)

Mr. Hutchinson presents in the above journal, (March 3, 1856,) a tabular report of twenty seven-cases, illustrating the connection between bronzed skin and diseases of the supra-renal capsules. Eleven of them are taken from Dr. Addison's volume. He then proceeds to examine, in some detail, the peculiar symptoms and events that attend the fatal cachexia, of which and of organic disease of the supra-renal capsules, the bronzing of the skin is, he thinks, really indicative. Mr. Hutchinson asserts:—

"That, notwithstanding the wide publicity which the theory has had given to it, and the great general interest which has been excited respecting it, the two following assertions may yet be made:—

"1. No single case has yet been recorded, in which a well-marked bronzed condition of the skin existed and recovery ensued, nor any in which, after death, the supra-renal capsules were proved to be healthy.

"2. No single case has yet been recorded in which, after death, both supra-renal capsules were found disorganized by chronic disease, and in which the skin during life had not assumed the bronzed condition."

In a note to the first of these assertions the author states, that in a "well-marked condition of bronzing" there ought to be some appearance of patches or mottling; and hence as wanting this characteristic, he is inclined "to ignore the seeming exception to the assertion furnished by case 3."

It is not enough, Mr. Hutchinson argues, for the arrest of function that parts of the supra-renal capsules, or even one of them be destroyed. If a considerable portion of their normal structure remains, their functions may be performed to such an extent as to prevent the manifestations of symptoms.

In less than three months after the announcement of the above dogmatic propositions by Mr. Hutchinson, an exceptional case was reported by Mr. Ogle at a meeting of the London Pathological Society, and is recorded in its Transactions, (vol. vii., 1856.) It was of a man, fifty years of age, who was brought into St. George's Hospital, laboring under ascites and anasarca. About ten weeks before entering the hospital he had suffered much from pain in the loins. This patient died in a paroxysm of dyspnœa. No kind of discoloration of the integuments was observable. On dissection, a large quantity of fibrous deposit was found within the substance of the supra-renal capsules and the kidneys; the renal and supra-renal veins were perfectly converted into fat. The supra-renal capsule was exceedingly large, owing to its extensive disorganization and occupation by soft deposits like that in the kidney. The abdominal viscera and lungs were congested; the heart soft and flaccid.

Dr. Fricke, of Baltimore, relates in this journal, for July, 1857, the outlines of a case of cirrhosis of the liver, and bronzed skin, but not so distinctly as to enable us to say to what extent or in what degree this color differed from or was confounded with that of jaundice, which latter is described in the advanced stage of the disease, or about three months before death, as universal. "As the case progressed, the principal phenomena exhibited were dropsy, hemorrhage, black urine, and bronzed skin." This last symptom was first observed in January—the patient died on April 29th, 1857. "At the commencement it was not well defined nor more decided than is often observed in cases of cirrhosed liver, but, after a time, became so deep as to attract decided attention. It was limited to the forehead, face, and neck, and almost as deep in hue as is found in the livers of remittent fever patients. One fact is worthy of notice. The demarkation between this color and that of the rest of the body was not clearly defined, but they merged into each other by degrees." The autopsy was made by Dr. Fricke, aided by Dr. Johnston. "Bronzed hue not so well marked, jaundice universal, and of a deep lemon tint, emaciation moderate, and legs œdematous. Heart and chest not examined. The abdominal cavity contained about one gallon of serous fluid, and the intestinal canal was filled with dark liquid blood. Kidneys congested and somewhat enlarged, otherwise healthy. Supra-renal capsules of natural size, hue, and consistence; no alteration whatever observable." The liver was much smaller than natural, and of a bronzy hue externally—surface irregular and nodulated.

Dr. Harley, whose experiments and views respecting the physiology of the supra-renal capsules we have freely introduced in the present argument, for such our readers will see is the stage the subject has reached, enters on the question of the pathology of these glands. Here he is again in the opposition, but with no caviling spirit. He strengthens his position with a goodly array of facts, which we shall now briefly notice. After adverting to the state of the question as it had been presented by Dr. Addison, Mr. Hutchinson, and others, and as may be learned from preceding pages of this article of ours, Dr. Harley tells us that "at the present moment the case is very different; several cases have been reported in various journals where extensive and even total destruction of the supra-renal capsules existed, unaccompanied by any discoloration of the skin. In proof of this, I may cite the following twelve examples." We introduce them here in an abbreviated state. Professor Friedreich* mentions two cases of amyloid degeneration of the supra-

* Virchow's Archives, April, 1857, p. 389.

renal capsules, in which there was no bronzing or change of color in the skin. Mr. Davis,* of Bath, at one of the meetings of the Bath and British Association, reported two cases of extensive disease of the supra-renal capsules, without any bronzing of the skin. Professor Virchow writes to Dr. Harley, that he has met with several cases of tubercular deposit in one or both supra-renal capsules, and one case in which both capsules were destroyed by cancerous deposit, but without any bronzing or discoloration of the skin having been observed in any of them. An interesting case of this nature is described in Jones and Sieveking's *Pathological Anatomy.* Dr. Harley has himself examined one case of complete degeneration of the supra-renal capsules, without any bronzing of the skin having been noticed. Of similar purport is a case mentioned by Dr. Klob, assistant to Rokitansky, in a man whose skin and face had undergone no change in color. Reference has been made already to the case reported by Dr. Ogle. A case of cancer of the supra-renal capsules and of the other organs, with no discoloration of the skin, is reported by Drs. Peacocke and Bristowe, in the *Transactions of the Pathological Society,* (vol. vii.) They describe another case of the same kind—cancerous capsules and no discoloration. A very curious case is reported by Professor Martini, of Naples. It is that of a man aged forty, the father of three children; he died of phthisis. His skin was remarkably fair for an Italian; and yet, on *post-mortem* examination, the supra-renal capsules were found entirely wanting.† Dr. Brittan (*Brit. Med. Journ.*, February 6, 1858,) relates two cases of supra-renal capsular disease, in which no bronzing of the skin occurred. "A number of cases have been reported in the journals, where only one supra-renal capsule was diseased, (see cases mentioned by Ogle, Hutchinson, Wilks, Murchison, and others,) and where bronzed skin was sometimes present, sometimes absent; but I refrain from mentioning them, as they throw no light upon the subject."

Here Dr. Harley closes the negative answer to the question—"Is disease of the supra-renal capsules invariably accompanied by bronzed skin?" He next proceeds to a consideration of the other question propounded by him, namely, "Is bronzed skin always associated with disease of the supra-renal capsules?" We shall follow him in this line of argument.

M. Puech communicates the case of a man, aged fifty-four, whose skin was observed to become gradually darker in color for a year and a half before his death. "The change of tint was accompanied by loss of strength, bad digestion, alternate diarrhœa and constipation. During a

* British Medical Journal, February 28, 1857.

† Comptes Rendus, December 1, 1856.

short interval he slightly improved, but afterwards he was seized with pain in the right iliac region, followed by black fetid stools. He subsequently died of peritonitis, from inflammation of the intestines. In this case, the bronzing of the skin was most decided upon the face, breast, abdomen, and anterior and interior of the thighs." Contrary, however, to the expectations of M. Puech, and others who saw the patient, the supra-renal capsules, after being minutely examined, did not exhibit the slightest alteration. Dr. Harley next quotes an abtract of Dr. Fricke's case, noticed by us in a preceding paragraph. Dr. Linton, Professor Simpson, Mr. George May, Mr. Hutchinson,* and Professor Virchow are named by Dr. Harley as relators of cases in which the skin was bronzed, and the renal capsules in a normal condition; and as far as their testimony goes, the second question propounded by this gentleman meets with a negative reply, and he deems himself justified in saying that discoloration of the skin is not always associated with supra-renal capsular disease. He adduces, in further confirmation of this view, the case reported by Dr. Sloane.

In speaking of the causes of bronzed skin, Dr. Harley draws from natural history and comparative physiology interesting instances of the difference of color of the skin in certain animals caused by modifications of light and season, acting, however, on constitutional peculiarities. The long retention of these peculiarities, under great difference of climate and atmospherical exposure, is observed among the different races of mankind. "Natural physiological changes occurring at different periods of life, also react upon the cutaneous function." The approach of puberty, and still more pregnancy, are examples to the point. Disordered chromatogenous function may exist without any disorder of the general health. "I might make mention," remarks Dr. Harley, "of other forms of discolored skin—such, for example, as the *melanopathia syphilitica, chloasmæ,* or *maculæ hepaticæ,* and even that which comes from prolonged exposure to the heat of charcoal, 'Ephelis ignealis,' of French authors; but I think that the cases I have already cited are sufficient to show that partial or even general discoloration of the skin occurs under such a variety of circumstances that it can scarcely be regarded as depending upon any one particular form of disease."

Under the microscope, a piece of bronzed skin taken from a patient whose renal-capsules were diseased, presents the very same appearances as the skin of the negro. "The pigmentary matter was deposited in the epithelium cells of the under layer of the epidermis—the rete mucosum, as it is sometimes called. The superficial layer of the epidermis, on the other hand, was almost entirely free of pigment." As a general rule,

---

* Transactions of the Pathological Society, vol. vii. p. 341.

† Medical Times and Gazette, August 29, 1857.

"the coloring matter was not limited in its distribution to the nucleus, but spread throughout the cell. The nucleus was, however, more deeply tinged." Dr. Harley has also examined bronzed skin taken from a patient with healthy supra-renal capsules, and convinced himself that its microscopical structure was in every respect identical with that taken from the other patient whose supra-renal capsules were diseased.

We need not repeat Dr. Harley's creed respecting the relations of bronzed skin to diseased renal-capsules. It is inferrible plainly enough from all that we have introduced in his line of argument and illustrations. On the anatomical and physiological grounds already stated, he looks upon "the symptoms of anæmia, languor, debility, feebleeness of the heart's action, and irritability of the stomach, not as the result of the suppression of the functions of the supra-renal capsules, but rather as being occasioned by a diseased state of the solar plexus *per se*, or by an irritation of the ganglionic system of nerves caused by the sole proximity and intimate connection of diseased supra-renal capsules."

We now revert to the propositions laid down by Mr. Hutchinson, repeated on p. 208; but it is not necessary to point out the extent to which they have been weakened, if not rebutted, by the opposing counsel in the person of Dr. Harley. Before going any farther, let us, however, give Mr. Hutchinson, or the side he advocates, the benefit of testimony additional to that adduced by him in his tabular report of twenty-seven cases of bronzed skin and disease of the supra-renal capsules. Cases of the like nature have been reported by Dr. Baly in a lad aged eighteen years;* Dr. Adam Martin in a married female aged fifty years;† Dr. Tyndale Robertson in a laborer aged twenty-six years;‡ Dr. Edward Gibbon in a cabman aged fifty-two years;§ M. Trousseau in a coachman aged thirty-seven, a patient at the Hotel-Dieu, (Paris;) M. Malheur in a woman aged forty-eight years, at the Hotel-Dieu, (Nantes;) M. Cazenave in a man of intemperate and otherwise vicious habits, in the Hospital of St. Louis, (Paris;) Dr. Christie in a woman aged thirty-six years; W. C. in three cases of advanced phthisis;|| M. Sécond Feruel;¶ Dr. Addison in a female eighteen years of age, whose skin was darkened, almost blackened; Dr. Isaac E. Taylor in two cases at the Bellevue Hospital, New York; the first, a male laborer, aged twenty-two; the second, a male, aged forty-two. Of the above fifteen cases the connection between bronzed skin and diseased supra-renal capsules was demonstrated by *post-mortem* examinations. The same cannot be said of eight of the twenty-seven cases in Mr. Hutch-

* Transactions of the Pathological Society of London, vol. viii.

† Brit. Med. Journ., May 15, 1858.

‡ Medical Times and Gazette, vol. xii.

§ Ibid, vol. xiii.

|| Ibid, for the preceding seven cases.

¶ Gazette Medicale, Oct. 4, 1856.

inson's tabular report, as of them no autopsy was made. Two were still living,—one reported as a case of recovery.

The interest which is attached to the discolorations of the skin, in the form of cachexia with anæmia, described by Dr. Addison, depends on the value of this appearance as an aid to diagnosis, which, considering the usually rapid course and fatal termination of the disease, ought to be made as early and with as much certainty as possible. In completion of what we quoted from that gentleman on this point, we shall now introduce the observations of Mr. Hutchinson and Dr. Taylor. The term "bronzing" was first used by Mr. Hutchinson. The color of the skin when thus discolored resembles strikingly that of a bronzed statue from which the gloss has been rubbed off. Pressure has no effect in causing its diminution. Dr. Taylor is of opinion that this hue is only characteristic of the more advanced stage of the disease; and even here, he thinks, the comparison of color would be more appropriate if one were to take for its subject a mulatto, or preferably, a West Indian. The shades described by Dr. Addison, in page 206, are quite characteristic when the disease assumes a chronic character; but, in the early stage, Dr. Taylor compares the discoloration to that caused by long exposure to the sun, and increasing to a darker hue. "It seems as a rule," according to Mr. Hutchinson's observations, "to commence first in patches with ill-defined borders, on those parts most exposed to the sun and to friction, the neck, the backs of the hands, the front of the thighs, the arms, etc. Around the nipples, and in other parts where pigment naturally abounds, it is generally well marked, while others, possessing little or no pigment originally, as in the palms of the hands, the ungual matrices, etc., remain as pale as ever. This tendency to show itself in patches, is strongly in support of the belief that the change is really one of deposit of pigment, which derives confirmation from the circumstance that, in not a few cases, the punctate or even patchy deposit of black matter was obscured in the mucous membrane of the mouth, and in the serous investments of the abdominal viscera." The author attaches great importance, as a pathogonomic sign, to the presence of blood stains on the mucous linings of the lips. In one of Dr. Taylor's cases, the line of demarkation between the face, which was discolored, and the scalp and ears, which were not affected, was very distinct; as was also the pigment on the lips.

The paleness of the conjunctiva, and of the matrices of the nails, in this disease, are in strong contrast with the yellow hue of the same parts in jaundice, looking to its general symptoms and the uniform diffusion of its peculiar tint. A sunburnt skin will be known by its appearing only on parts which are habitually exposed. "Patches of *pityriasis versicolor* sometimes remarkably resemble those of bronzed skin. Their limitation to the abdomen and chest, their defined outline, their furfuraceous surface, the slight itching which attends them, their contagious character, and, above all, the microscopic examination of the cuticle, furnish, however, abundant means by which to distinguish between the two." With common care the diffused brown muddiness of some forms of periodical fever, and of malignant cachexia, may be distinguished from the bronzed skin.

An examination of the skin, in one of Dr. Taylor's cases, was made by Dr. J. C. Dalton, Jr., with results analogous to those which are described by Dr. Harley at pp. 211–12. Dr. Dalton says, that the coloring matter was deposited in a granular form, and that it could not be distinguished from the pigment of a mulatto.

Among the symptoms which, next to bronzed skin, are to aid us in our diagnosis of the disease now before us, Mr. Hutchinson mentions *debility*, which manifests itself in an extreme degree, without any evidence of ordinary organic affection, or special functional disturbance. *Emaciation.*—The want of correspondence between the extreme debility and loss of flesh is one of the features of the disease. Dr. Addison's observation, that flabbiness of the solids rather than actual wasting, is the actual state of the patient, applies to the majority of cases. *Anæmia.*—In nearly all the cases there was an evident deficiency of the colored constituents of the blood; and in the two in which this fluid was examined under the microscope, it was found to be loaded with white corpuscles. "To the impoverished state of the blood is no doubt to be referred the breathlessness in exertion, the debility, the feebleness of the heart's action, and perhaps the irritability of the stomach." *Pulse.*—Its chief peculiarity consisted on its extreme softness and compressibility. *Tongue.*—It presents no appearance different from that met with in most states of debility. *Dyspepsia.*—In a majority of the cases great irritability of the stomach was present, marked by nausea and vomiting, and accompanied with a sense of pain, and sinking of the epigastrium. For the most part there was constipation, and in some instances diarrhœa. Often the patients had been pronounced to have had bilious attacks. *Urine.*—Although searches were made for the purpose, neither sugar nor albumen was detected. *Lumbar Pains.*—Aching, more or less severe, in the back and loins, was a symptom present in a considerable proportion of the cases; although in two of these there was disease of the vertebræ. "*Nervous Symptoms, Convulsions*, etc.—Symptoms referable to disorder of the cerebro-spinal functions occurred in several of the cases. In three, epileptiform convulsions preceded death. In one, failure of memory, and remarkable change in temper, was observed; and in a second, numbness of the fingers, legs, and the tip of the tongue, had been present. In one, the man had suffered from tic douloureux." It will not be uninteresting or uninstructive to connect these symptoms of great derangement of the nervous system with analogous disturbances in animals after ablation of the supra-renal capsules, as practiced and recorded by Dr. Brown-Séquard. *Odor of the Body.*—Dr. Addison makes no allusion to this symptom, which was noticed in two of the cases tabulated by Mr. Hutchinson. In one case there was a very offensive odor given out from the body for a few weeks, and, in the other, only for a few days previous to death. In this latter the odor was less unpleasant from that portion of the skin covered with clothing than from the exposed surface, as for instance, of the neck.

Much yet remains to be learned to complete the diagnosis of the disease of the supra-renal capsules, or of the contiguous nervous plexuses affected by their lesion. Dr. Addison, at a meeting of the London Medico-Chirurgical Society,* in describing a remarkable case which he had seen of discoloration of the skin amounting almost to blackness, in a young lady aged eighteen, associated with extensive lesions of the supra-renal capsules, expressed his entire belief that the disease proceeded from the morbid change of these organs alone. He added, and his remarks must be received as highly suggestive: "We know, however, that these organs were situated in the direct vicinity and in contact with the solar plexus and semilunar ganglion, and received from them a large supply of nerves; and who could tell what influence the contact of these diseased organs might have on these great nervous centres, and what share this secondary effect might have on the general health and on the production of the symptoms presented?" Dr. Addison, on the same occasion, disclaimed attaching any importance to the discoloration of the skin. He believed that the disease might occur, and had occurred, without any change of this organ.

As regards the "mode of death," speaking generally, "we may say that the phenomena attending death are those of utter prostration of the vital powers, not unfrequently complicated with disturbance of the nervous functions."

The prognosis in a case of true bronzing of the skin is always unfavorable. After the expression of such an opinion, little can be expected from treatment. There is reason for believing, however, that various as may be the morbid textural changes of the supra-renal capsules, they may be supposed for the most part to have originated in or to have been closely allied to inflammation. Were we to admit this hypothesis, we should be justifiable in using such means as are believed to possess an influence over that process. Mr. Hutchinson suggests the use of mercury in small doses, or that of the iodide of potassium, while the patient's strength is in the mean while supported by nutritious but non-stimulating food.

---

* Reported in the Medical Times and Gazette, February 20, 1858.

The *morbid anatomy* of the supra-renal capsules, investigated by Mr. Hutchinson, shows: 1. Acute and recent inflammation, ending in abscess, in one case. 2. Atrophy, with fibro-calcareous concretions, in seven cases. 3. Conversion of the organs into a kind of fibroid structure, with great enlargement and induration, with entire loss of healthy tissue, in two cases. 4. The deposit of tubercle in three cases, and, coincidently, great enlargement of the organ. 5. Cancer in six cases. "In all it was secondary to the same disease in other organs. In four it affected but one organ, and in two both were involved. In but one case was complete disorganization of both affected." A remarkable symmetry of disease appears to have existed in all except in the cases of cancer.

Serious blanks are yet to be filled up in the history of the supra-renal capsules. We are ignorant of their functions; nor are we able to speak in decisive terms of the varieties of appearances compatible with health; and we are entirely at a loss for the etiology of their diseases. Future observers can hardly be too minute in their description of the actual symptoms of the disease which is marked by discolored or bronzed skin, nor of the complaints and all the circumstances preceding it. Especially is it desirable to improve the diagnosis so far as to enable the practitioner to ascertain the existence of the supra-renal capsular disease in cases in which there is no discoloration, or at a stage antecedent to the appearance of this change in the skin.

